# A shared feature between the salient distractor and target turns early quitting effect to delayed quitting effect when the target is absent

**DOI:** 10.1186/s41235-025-00677-8

**Published:** 2025-09-24

**Authors:** Wenjie Peng, Yujun He, Xinyu Shi, Jie Yuan

**Affiliations:** 1 School of Psychology, South China Normal University, Guangzhou, 510631 Guangdong China; 2 School of Education (Shanwei), South China Normal University, Shanwei, 516600 Guangdong China

**Keywords:** Visual search, Early quitting effect, Salient distractor, Target-distractor similarity, Attention suppression

## Abstract

In a seminal paper, Moher (Psychol Sci 31(1):31–42, 10.1177/0956797619886809, 2020) reported that a salient distractor induced observers to quit the search early when the target was absent and increased the error rate when the target was present. This early quitting effect (EQE) was considered to impact real-world target detection. We were interested in how the EQE would be influenced when the similarity between the target and the salient distractor increased. This may more closely resemble real-world situations and may reveal underlying mechanisms of the EQE, as increased similarity could either raise costs of attention suppression, leading to the disappearance or even reversal of the EQE, or trigger the sense of effort in searching, resulting in the appearance of the EQE. Through two experiments, we demonstrate that the effect of a salient distractor on detecting a target was limited by the similarity of the target and the salient distractor. In Experiment 1, we conducted a task with a salient distractor that differed in color, size, and orientation from the target to replicate the EQE. We found that participants reacted faster in target-absent trials and less accurately in target-present trials, thus validating the experiment. However, when the similarity of the salient distractor and target was increased by sharing the same orientation feature in Experiment 2, the EQE did not occur. Specifically, regardless of target presence, a salient distractor delayed the search time and did not influence the error rate. These findings support that attention suppression, rather than the sense of search effort, is a subprocess of the EQE.

## Introduction

Individuals often try to focus attention on target information during visual search, but sometimes attention is drawn to distractions against their intention. Researchers study this attentional capture phenomenon using a singleton search task in which observers are required to search for a predefined target item among a series of similar nontarget items (Theeuwes, 1991). The target item appeared in all trials, while in some cases, one of the nontargets was transformed into a highly salient and task-irrelevant distractor. A typical finding was that the presence of a salient distractor increased the time required to find a target (Geyer et al., 2008; Hickey et al., 2006; Theeuwes, 1992).

Considering that targets are not always present in real-world visual searches, Moher (2020) introduced a new target-detection task to further study how salient distractors influenced visual search in both target-present and target-absent conditions. In this task, participants were required to determine whether a vertically oriented blue rectangle target was present among tilted blue rectangles. A salient distractor, presented as a larger, red, and tilted rectangle with a delayed onset, appeared in half of the trials. It was found that the presence of a salient distractor shortened search time in target-absent trials and increased the error rate in target-present trials. In other words, salient distractors may lead to early quitting when searching for the target, thereby increasing the likelihood of target misses. It was a counterintuitive and seminal finding challenging the widespread belief that distractors slow down visual search.

Recently, this so-called *early quitting effect* (EQE) has been studied in various search contexts to explore the boundary of the effect of the distractor. An eye-tracking study found that the occurrence of the EQE was related to the amount of information that observers extract from the task (Shaikh et al., 2025). Salient distractors caused observers to have fewer eye movements and search a smaller area of the display before they responded that the target was absent and increased the error rate caused by not fixating or recognizing that there was a target. This EQE remained regardless of factors such as target prevalence (Moher, 2020), delayed onsets of the distractor (Lawrence et al., 2023a, 2023b), or whether emphasizing responder speed or accuracy (Lawrence et al., 2023a, 2023b). In addition to the entirely task-irrelevant salient distractor, a misleading but task-relevant, salient cue could also trigger the EQE when the cue was largely reliable in highlighting a target (Moher et al., 2024). However, the EQE disappeared when the distractor was more difficult to reject, such as when it was less salient (Lawrence & Pratt, 2022), of low prevalence (Lui et al., 2024), or matched the content of visual working memory (Wu & Pan, 2022). Notably, in Lawrence and Pratt’s (2022) study, the distractor was low in salience but similar to the target in size. In contrast, Lui et al. (2024) used a distractor that was highly salient due to its low prevalence but dissimilar to the target. These studies suggested that target-distractor similarity might influence the EQE, yet whether this similarity contributed to the disappearance of the EQE remains uncertain.

A recent study showed that, when the target was absent, a salient distractor sharing the same directional features as the target led to longer search times compared to when there was no salient distractor (Becker et al., 2025). The study suggests an effect of target-distractor similarity and uses a drift diffusion model to explain it. Building on these, our study seeks to further investigate the psychological implications of this effect.

Target-distractor similarity has been identified as an important factor guiding our attention and influencing how fast observers search the target in a visual search task (Chapman & Störmer, 2022; Duncan & Humphreys, 1989; Wolfe & Horowitz, 2004). In many real-world search scenarios, salient distractors are often highly similar to the targets. For example, in daily life, parents need to find their child from a group of children about the same age after school. When screening airport baggage, target dangerous objects (e.g., folding knives) can have a similarity in shape and density with nontarget but salient everyday objects (e.g., metal hairpins). As such, understanding how the target-distractor similarity would influence the EQE contributes to advancing our knowledge of how a salient distractor that more closely resembles real-world situations influences visual search.

The role of target-distractor similarity may provide insight into the underlying mechanisms of the EQE, especially in relation to how the salient distractor influences cognitive processes. On one hand, the salient distractor may initially capture attention (Forschack, et al., 2022; Lawrence et al., 2023a, 2023b; Lui et al., 2024). In the target-absent condition, because the distractor is completely distinct from the target, individuals can suppress distractor processing or withdraw attention with high certainty, quickly excluding the distractor and focusing on verifying other nontargets. Thus, a salient distractor reduces the complexity of search compared to when no salient distractor is present, leading to the EQE. If the target was similar to the distractor, the salient distractor cannot be quickly excluded, thus increasing cognitive cost and interfering with attention. As a result, fixation durations on the salient distractor and response time to the target may be longer. In this assumption, when the target was completely different from the distractor, the EQE would appear. However, target-distractor similarity may lead to longer search times in target-absent trials, causing the EQE to disappear or even reverse, similar to the findings of Becker et al. (2025). On the other hand, according to Moher (2020), the EQE may result from a salient distractor increasing the sense of effort in the search task by interacting with attention resources. The presence of a salient distractor may occupy attention resources and play a psychological “placebo” role, making participants feel they have exerted enough effort in the search and prompting them to quit early in target-absent trials. In this assumption, regardless of the similarity of the target and salient distractor, the EQE would appear.

In sum, the current study aimed to replicate and expand upon the finding of the EQE by exploring how target-distractor similarity influences the role of the salient distractor in search time and error rate under both target-absent and target-present conditions. Specifically, in Experiment 1, based on Moher (2020) and Lawrence et al., (2023a, 2023b), we conducted a target-detection task in which the salient distractor was different from the target in three features: color, size, and orientation. This experiment was expected to replicate the EQE when the target was dissimilar to the salient distractor. In Experiment 2, the only difference from Experiment 1 was that we used a distractor that shared the same orientation as the target, thereby increasing its similarity with the target but decreasing its similarity with other nontarget filler items. We want to see whether the result of Experiment 2 would differ from those of Experiment 1 to further explore the mechanisms of the EQE.

## Materials and methods

### Experiment 1

#### Participants

Two previous studies on the EQE showed that the interaction between distractor presence and target presence condition, which we were most interested in, has an effect size of at least $$\eta_{{\text{p}}}^{2}$$ = .19 (Lawrence & Pratt, 2022; Moher, 2020). Thus, assuming a within-factors ANOVA with effect size of $$\eta_{{\text{p}}}^{2}$$ = .19, power of .95, and an alpha level of .05, the required sample size should be at least 13 participants according to G^*^Power 3.1.9.7 (Faul et al., 2009). Considering the possibility that some participants may fail the screening criteria, in Experiment 1, 20 students (7 males, *M*_age_ = 20.4, SD_age_ = 1.96) were recruited from South China Normal University. According to similar visual search studies, this sample size was adequate to yield reliable research outcomes (Bhat et al., 2024; Foerster & Schneider, 2020). All participants were right-handed, with normal or corrected-to-normal vision, and no color blindness was reported. They gave informed and written consent for their participation and received approximately $1 as compensation. This study was approved by the ethical committee of South China Normal University.

#### Stimuli and apparatus

**Fig. 1:**
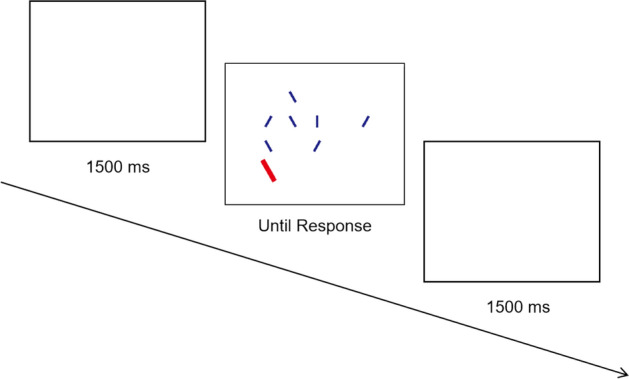
Flowchart of procedure for Experiment 1. In this example trial, a target (small, blue, vertical rectangle) and a salient distractor (large, red, tilted rectangle) are present, and the trial includes eight items

The visual stimuli were generated by E-Prime (version 2.08) and displayed on a liquid crystal display monitor (refresh rate: 60 Hz; resolution: 1,024 × 768 pixels) in the laboratory. The stimuli included targets, fillers, and salient distractors presented on a white background (see Fig. 1). The target item was a blue, vertically oriented, and 40 pixels × 8 pixels rectangle (RGB: 29, 32, 136). All other nontarget items were randomly tilted 30 degrees to the right or left. The filler items were the same size and color as the target, and the salient distractor was a large, red, and 80 pixels × 16 pixels rectangle (RGB: 230, 0, 18). There were two set sizes in the experiment: four items and eight items. For the four-item trials, items randomly appeared within a grid of 320 × 320 pixels. For the eight-item trials, items randomly appeared within a 400 × 400 pixels grid. In both conditions, there were 50 pixels of spacing between the grid lines, and no items could appear in the same location or overlap with each other.

#### Procedure

In each trial, participants were asked to indicate whether a target was present (by pressing the “z” key) or absent (by pressing the “m” key) as quickly and accurately as possible. The target was randomly presented in 50% of the trials. A salient distractor also appeared in half of the target-present trials and target-absent trials. As a result, all conditions have the same possibility to occur. The target and distractor were counted in the total number of items in a trial. That is, regardless of whether there was a target or a distractor, the search size was still four or eight. The search array remained on the screen until the participant responded.

Each participant completed 216 trials, including 16 practice trials and two blocks of 100 experimental trials. The practice trial was followed by 1-s feedback. This feedback was not given after completing experimental trials and was replaced with a 1.5-s blank intertrial interval. Participants took a break at the end of each block, and the experiment took 10 min to complete.

Notably, in the original experiment, a delayed onset was used to make the distractor as salient as possible in trials when the salient distractor was present (Moher, 2020), but Lawrence et al. (2023a, 2023b) have shown that the delay onsets were not necessary. Following this finding, the distractor appeared at the same time as the onset of the search array in Experiment 1.

#### Results

A 2 × 2 × 2 repeated-measures ANOVA was conducted with target presence (present vs. absent), distractor presence (present vs. absent), and set size (four vs. eight items) as factors on measures of response times (RT) and error rate. All participants had an overall error rate below 40%, with error rates in each condition not exceeding 90%, thus meeting the error rate criteria of the previous EQE studies (e.g., Moher, 2020) and were included in the final analysis. RT measures were based only on trials with correct responses. Trials with RTs below 200 ms or over 10 s were removed from the analysis, and RTs exceeding ± 2.5 standard deviations of the mean of each condition for each participant were excluded. See Tables 1 and 2 for descriptive statistics and a full report of the ANOVA results of Experiment 1.

**Table 1:** Descriptive statistics for Experiment 1

Dependent variable and target presence	Distractor	Set size	
		Four items	Eight items
		*M* (SD)	*M* (SD)
Response time (ms)			
Target present	Present	686 (160)	891 (265)
Target present	Absent	647 (130)	839 (236)
Target absent	Present	740 (202)	1118 (419)
Target absent	Absent	797 (228)	1154 (444)
Error rate (%)			
Target present	Present	11.00% (9.35%)	12.40% (8.40%)
Target present	Absent	4.40% (4.66%)	9.20% (8.12%)
Target absent	Present	1.00% (2.20%)	0.80% (1.64%)
Target absent	Absent	1.40% (2.68%)	1.80% (3.30%)

**Table 2:** Results of the repeated-measures ANOVA for Experiment 1

Analysis and dependent variable	*F* (1,19)	*p*	$\eta_{{\text{p}}}^{2}$
Set size			
Response time	49.86	< .001	.72
Error rate	8.94	.008	.32
Target presence			
Response time	32.87	< .001	.63
Error rate	31.18	< .001	.62
Distractor presence			
Response time	< .01	.983	< .01
Error rate	11.99	.003	.39
Set size × target presence			
Response time	42.06	< .001	.69
Error rate	5.52	.030	.23
Set size × distractor presence			
Response time	1.37	.256	.07
Error rate	2.32	.144	.11
Target presence × distractor presence			
Response time	24.32	< .001	.56
Error rate	22.04	< .001	.54
Set size × target presence × distractor presence			
Response time	.10	.756	.01
Error rate	1.48	.239	.07

##### Response times

The first set of analyses validated whether data collection in the laboratory had the same standard visual search effects as through the online environment used in the target-discrimination search task (e.g., Lawrence et al., 2023a, 2023b; Moher, 2020). The results showed that RTs were significantly shorter on target-present trials (*M* = 766 ms, SD = 193 ms) than target-absent trials (*M* = 952 ms, SD = 319 ms), *F* (1, 19) = 32.87, *p* < .001, $\eta_{{\text{p}}}^{2}$ = .63. RTs were shorter for four-item trials (*M* = 718 ms, SD = 175 ms) relative to eight-item trials (*M* = 1,001 ms, SD = 337 ms), *F* (1, 19) = 49.86, *p* < .001, $\eta_{{\text{p}}}^{2}$ = .72. There was no main effect of distractor presence, *F* (1, 19) < 1,* p* = .983. Moreover, there was a significant interaction between target presence and set size, *F* (1, 19) = 42.06, *p* < .001, $\eta_{{\text{p}}}^{2}$ = .69, and a significant interaction between distractor presence and target presence, *F* (1, 19) = 24.32, *p* < .001, $\eta_{{\text{p}}}^{2}$ = .56. All other interactions were nonsignificant, *p*s > .1. These basic results were consistent with previous findings of Moher (2020).

**Fig. 2:**
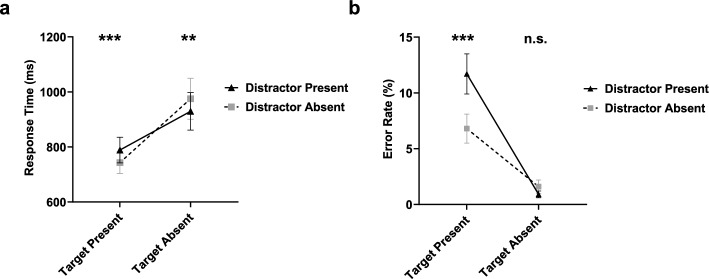
Mean **a** response time and **b** error rate for distractor-present/absent conditions in target-present/absent trials in Experiment 1. Error bars represent the standard error of the mean for response time or error rate. ***p < .001; **p < .01, n.s. *p* ≥ .05

The primary interest in this experiment was the relationship between distractor presence and target presence condition. Simple effect analyses were conducted to compare RT differences between the distractor-present and distractor-absent conditions in both target-present and target-absent trials. When targets were present, RTs were significantly longer when distractors were also present (*M* = 789 ms, SD = 208 ms) than when distractors were absent (*M* = 743 ms, SD = 180 ms), *F* (1, 19) = 19.54, *p* < .001, $\eta_{{\text{p}}}^{2}$ = .51. It indicated that the distractor captured attention and, as a result, increased the amount of time required to find the target. However, when targets were absent, the opposite pattern emerged. RTs were shorter on distractor-present trials (*M* = 929 ms, SD = 305 ms) than distractor-absent trials (*M* = 975 ms, SD = 334 ms), *F* (1, 19) = 14.68, *p* = .001, $\eta_{{\text{p}}}^{2}$ = .44 (see Fig. 2). In a word, salient distractors that appeared simultaneously with other search items compared with those of absence still triggered the EQE and decreased the time of quitting the search, thus validating those findings reported in previous studies (Lawrence et al., 2023a, 2023b; Moher, 2020).

Finally, we ran two Bayesian paired-sample *t* tests to assess the effect of distractor presence on RT. Based on our assumptions and the findings of previous studies (Lawrence et al., 2023a, 2023b; Moher, 2020), we set different hypotheses in the target-present and target-absent trials. When the target was present, the null hypothesis was that distractor presence did not influence RTs, and the alternative hypothesis was that RTs were longer in distractor-present trials than in distractor-absent trials. When the target was absent, the alternative hypothesis became that RTs were shorter in distractor-present trials than in distractor-absent trials. There was strong evidence in favor of the alternative hypothesis in the target-present (*BF*_10_ = 214) and target-absent conditions (*BF*_10_ = 65.83). That is, distractors increased the search time when the target was present but decreased the search time when the target was absent.

##### Error rates

Error rates were higher when distractors were present (*M* = 6.3%, SD = 4.4%) than when they were absent (*M* = 4.2%, SD = 3.2%), *F* (1, 19) = 11.99, *p* = .003, $\eta_{{\text{p}}}^{2}$ = .39. There was a significant interaction between distractor presence and target presence condition, *F* (1, 19) = 22.04,* p* < .001, $\eta_{{\text{p}}}^{2}$ = .54. To better understand this interaction, we also conducted simple effect analyses comparing error rates between distractor-present and distractor-absent conditions for both target-present and target-absent trials. When targets were present, error rates were higher in the distractor-present condition (*M* = 11.7%, SD = 8.1%) than distractor-absent condition (*M* = 6.8%, SD = 5.6%), *F* (1, 19) = 20.56, *p* < .001, $\eta_{{\text{p}}}^{2}$ = .52. In other words, when a distractor was present, participants were more likely to miss a target. However, when targets were absent, errors were quite low overall (*M* = 1.3%, SD = 1.7%), and there was no main effect of distractor presence, *F* (1, 19) = 1.75, *p* = .201.

We ran two Bayesian paired-samples *t* tests to examine the effect of distractor presence on error rate in both the target-present and target-absent trials. The null hypothesis was that distractor presence had no effect on error rate and the alternative hypothesis was that error rates were higher in distractor-present trials than in distractor-absent trials. Analysis for target-present trials provided strong evidence in favor of the alternative hypothesis, *BF*_10_ = 268.78. For target-absent trials, the analysis favored the null hypothesis, *BF*_10_ = 0.11. These findings replicated the early quitting effect of the salient distractor on target misses.

### Experiment 2

#### Participants

Twenty students (2 males, *M*_age_ = 20.85, *SD*_age_ = 2.30) from South China Normal University participated in Experiment 2. All participants were also right-handed, with normal or corrected-to-normal vision, and no color blindness was reported. They participated in a payment of approximately $1 and gave informed and written consent.

#### Stimuli

**Fig. 3:**
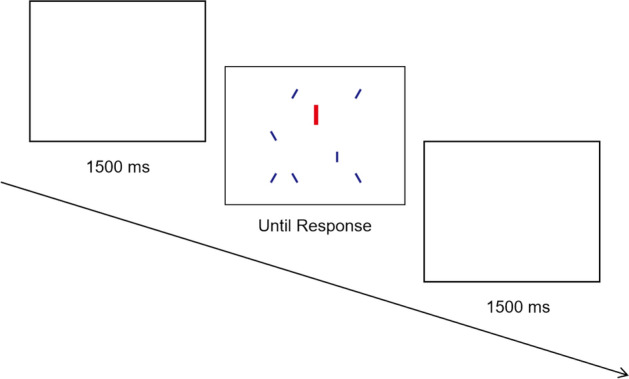
Flowchart of the procedure for Experiment 2. In this example trial, a target (small, blue, vertical rectangle) and a salient distractor (large, red, vertical rectangle) are present, with a total of eight items in the trial

The visual stimuli used in Experiment 2 were the same as in Experiment 1 except for the salient distractor. The shape, color, and size of the salient distractor in Experiment 2 were the same as in Experiment 1, but it was presented in a vertical orientation which is the same as the target and different from the other filler items (see Fig. 3). In other words, only the similarity between the salient distractor and target was increased.

#### Procedure

Identical to Experiment 1.

#### Results

A 2 × 2 × 2 repeated-measures ANOVA was conducted with target presence (present vs. absent), distractor presence (present vs. absent), and set size (four vs. eight items) as factors on measures of RT and error rate. Similar to Experiment 1, participants had an overall error rate under 40%, with no condition exceeding a 90% error rate, and were therefore included in the final analysis. Only trials with correct responses were considered for calculating response time (RT) measures. All trials with RTs below 200 ms or over 10 s were removed from the analysis, and RTs exceeding ± 2.5 standard deviations of the mean of each condition for each participant were excluded. Tables 3 and 4 show descriptive statistics and a full report of the ANOVA results of Experiment 2.

**Table 3:** Descriptive statistics for Experiment 2

Dependent variable and target presence	Distractor	Set size	
		Four items	Eight items
		*M* (SD)	*M* (SD)
Response time (ms)			
Target present	Present	765 (134)	1012 (204)
Target present	Absent	736 (117)	945 (237)
Target absent	Present	901 (196)	1352 (384)
Target absent	Absent	859 (214)	1316 (433)
Error rate (%)			
Target present	Present	6.00% (5.27%)	8.20% (7.95%)
Target present	Absent	6.20% (5.58%)	9.60% (5.57%)
Target absent	Present	1.00% (2.20%)	0.60% (2.68%)
Target absent	Absent	0.80% (2.09%)	0.40% (1.23%)

**Table 4:** Results of the repeated-measures ANOVA for Experiment 2

Analysis and dependent variable	*F* (1,19)	*p*	$\eta_{{\text{p}}}^{2}$
Set size			
Response time	87.45	< .001	.82
Error rate	4.17	.055	.18
Target presence			
Response time	40.98	< .001	.68
Error rate	53.05	< .001	.74
Distractor presence			
Response time	18.73	< .001	.50
Error rate	.14	.716	.01
Set size × target presence			
Response time	48.98	< .001	.72
Error rate	7.33	.014	.28
Set size × distractor presence			
Response time	.62	.441	.03
Error rate	.32	.577	.02
Target presence × distractor presence			
Response time	.12	.734	.01
Error rate	.43	.522	.02
Set size × target presence × distractor presence			
Response time	.82	.375	.04
Error rate	.38	.545	.02

##### Response time

Analysis showed that RTs were significantly shorter on target-present trials (*M* = 864 ms, SD = 163 ms) compared with target-absent trials (*M* = 1,107 ms, SD = 299 ms), *F* (1, 19) = 40.98, *p* < .001, $\eta_{{\text{p}}}^{2}$ = .68. RTs were shorter for four-item trials (*M* = 815 ms, SD = 157 ms) than eight-item trials (*M* = 1,156 ms, SD = 300 ms), *F* (1, 19) = 87.45, *p* < .001, $\eta_{{\text{p}}}^{2}$ = .82. Moreover, there was a significant interaction between target presence and set size, *F* (1, 19) = 48.98, *p* < .001, $\eta_{{\text{p}}}^{2}$ = .72. However, the three-way interaction between set size, distractor presence, and target presence was nonsignificant, *F* (1, 19) < 1, *p* = .375. These results were consistent with Experiment 1.

**Fig. 4:**
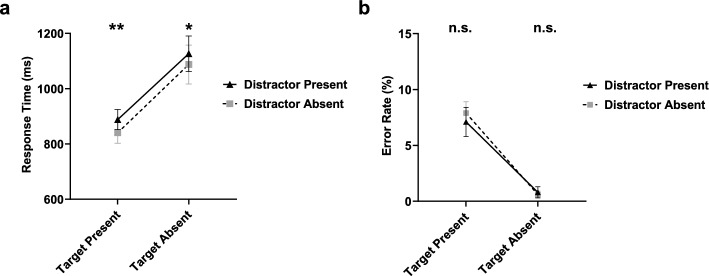
Mean **a** response time and **b** error rate for distractor-present/absent conditions in target-present/absent trials in Experiment 2. Error bars represent the standard error of the mean for response time or error rate. **p < .01; *p < .05, n.s. *p* ≥ .05

The primary variables of interest in this experiment were distractors with the same orientation as targets. There was a main effect of distractor presence on RTs, *F* (1, 19) = 18.73, *p* < .001, $\eta_{{\text{p}}}^{2}$ = .50. RTs were longer for distractor-present trials (*M* = 1,007 ms, SD = 217 ms) relative to distractor-absent trials *(M* = 964 ms, SD = 236 ms). However, there was no significant interaction between distractor presence and set size, *F* (1, 19) < 1, *p* = .441. Besides, there was no significant interaction between distractor presence and target presence,* F* (1, 19) < 1, *p* = .734 (see Fig. 4). These findings indicated that the increased similarity between salient distractor and target turns the early quitting effect into a delayed quitting effect when the target is absent.

Since we did not replicate previous findings that the salient distractor shortened search time when the target was absent, but instead found that the salient distractor lengthened RTs, we ran two Bayesian paired-samples *t* tests to test the effect of distractor presence on RT in both the target-present and target-absent trials. The null hypothesis was that distractor presence did not influence RTs and the alternative hypothesis was that RTs were longer in distractor-present trials than in distractor-absent trials. Analyses showed that moderate-to-strong evidence supported the alternative hypothesis in both the target-present trials (*BF*_10_ = 13.42) and target-absent trials (*BF*_10_ = 4.41). Thus, when the similarity between target and distractor increased, distractors prolonged the search time regardless of target presence.

##### Error rates

Different from Experiment 1, there was no significant main effect of distractor presence, *F* (1, 19) < 1, *p* = .716. The interaction between distractor presence and target presence was also nonsignificant, *F* (1, 19) < 1, *p* = .522.

Two Bayesian paired-sample *t* tests were conducted to further examine the effect of distractor presence on error rate in both the target-present and target-absent trials. The null hypothesis was that the error rate between distractor-present trials and distractor-absent trials was not different and the alternative hypothesis was that error rates were higher in distractor-present trials than in distractor-absent trials. The analysis favored the null hypothesis for target-present (*BF*_10_ = .16) and target-absent trials (*BF*_10_ = .31).

These findings suggest that the effect of a salient distractor on target misses disappeared after increasing the similarity of distractors to targets. In other words, the increased similarity between the salient distractor and target eliminated the EQE.

## Discussion

The current study investigated whether the early quitting effect (EQE), referring to a salient distractor that shortened target-absent RT and increased target-present error rate, would be influenced by the similarity between the salient distractor and the target. Overall, the role of the salient distractor varied depending on the similarity between the distractor and the target. In Experiment 1, we used a distractor that was completely different from the target to replicate the EQE. The EQE was triggered even without a delayed onset of the salient distractor and in a small group of observers, in line with those findings reported in previous studies (Lawrence et al., 2023a, 2023b; Moher, 2020; Shaikh et al., 2025). In contrast, in Experiment 2, when we only increased target-distractor similarity by sharing the same orientation feature based on Experiment 1, the EQE disappeared. The salient distractor delayed the search time and had no influence on the error rate in either the target-present or target-absent conditions.

These findings suggest that the EQE can be modulated by the similarity between the salient distractor and the target. They are consistent with those of Becker et al. (2025), who also conducted a target-detection task comparing conditions where a salient distractor either shared or differed in orientation from the target. Becker et al. (2025) explained the results using the drift–diffusion framework. According to the framework, when a distractor shared no features with the target, evidence quickly accumulated toward the decision that the target was absent. In contrast, a salient distractor caused a rapid accumulation of evidence toward the decision that the target was present when it shared features with the target. This led to the longer RTs when the distractor was similar to the target in the target-absent trials.

From a psychological perspective, the current study further clarifies the mechanism of the EQE by exploring how different distractors influence specific cognitive processes underlying behavioral performance. It provides evidence that the salient distractor can capture attention regardless of the target’s presence and opposes the assumption that the sense of effort prompts a shorter search time. Specifically, there are differences in the distractor functions within the search process when the salient distractor shares similarities with the target than when the salient distractor is distinct from it.

On one hand, the salient distractor may have two functions in the appearance of the EQE. First, it is a search item that provides a starting point. When a salient distractor is present, it captures attention before the target and other nontargets, aligning with the theory of stimulus-driven selection that attention is prioritized to shift toward salient stimuli regardless of task demands (Hickey et al., 2006; Theeuwes, 1991, 1992, 2025). Second, it signals that the distractor is easy to be excluded. The salient distractor’s large difference from the target allows individuals to be more certain that it is not the target. Therefore, in the target-absent trials, even though the salient distractor captures attention and requires cognitive resources for suppression, it enables individuals to quickly exclude the search item and to reduce the need for repeated searches. This allows for more efficient focus on verifying the remaining nontargets, ultimately reducing search complexity and leading to a shorter search time (i.e., the EQE). The findings of Shaikh et al. (2025) support this explanation, which showed that individuals had fewer fixations on the salient distractor after the first saccade when the salient distractor was present, suggesting less repeated search on the salient distractor after suppressing it. In addition, this exclusion process is fast and intuitive, which may incline individuals to believe that there is no target in the display after concluding that the distractor is not the target. As a result, in the target-present trials, when a salient distractor is present, individuals are more likely to miss the target.

On the other hand, when the salient distractor shares a feature with the target, it becomes both a starting point and a source of interference. The increased similarity between the distractor and target may make it more difficult to process the salient distractor by increasing the amount of information that observers need to compare, such as searching a larger area or repeatedly confirming the difference between the target and distractor. This additional cognitive effort to reject the distractor likely makes observers spend more time suppressing the salient distractor after attention was captured, and thereby slows the search speed. Moreover, it prevents individuals from making quick decisions about whether the distractor is the target or not, and they are less likely to develop the inclination that there is no target. Therefore, the difference in error rate between the distractor-present and distractor-absent conditions disappeared when the target was present. This mechanism of the EQE supports that the serial searches occur when the target is absent (Wolfe, 2020), at least the search from the salient distractor to the other nontargets is a one-by-one process. Future eye-tracking and electroencephalography (EEG) studies can investigate how eye movements and EEG signals change in the target-detection task when the distractor and target are similar to explore these possibilities.

An alternative explanation may be that the different findings in Experiments 1 and 2 were attributed to the distinction between searching for a feature and searching for an object. From this perspective, individuals only needed to search for a vertical feature as the target in Experiment 1, whereas in Experiment 2, they had to search for a blue and vertical target object. However, the evidence did not entirely support this possibility. According to the feature-integration theory (Treisman & Gelade, 1980), feature search is highly efficient. That is, increases in the number of items in the display have little effect on the time taken to respond when a target is present. By contrast, efficiency in searching for an object is lower, and as set size increases, response time increases markedly. The results of this study showed that RTs were significantly shorter for four-item trials relative to eight-item trials, in both Experiment 1 (*p* < .001) and Experiment 2 (*p* < .001). Therefore, individuals may search for the target as an object rather than a feature in these two experiments, even though the object was defined solely by its orientation feature in Experiment 1. The different findings between the two experiments cannot be fully concluded as a distinct search process between feature search and object search.

Some additional consequences from our manipulation of target-distractor similarity need to be noted. One consideration is that, as we examined the effect of target-distractor similarity across different experiments, individual differences might affect the reliability of our findings. Thus, future studies could use a within-subjects design to further compare the effects of varying target-distractor similarity within a single experiment, which would help to validate the robustness of this effect. Additionally, we found that overall RTs were longer in Experiment 2 compared to Experiment 1. It suggests that the overall difficulty may increase when the salient distractor is similar to the target, in line with previous visual search studies (e.g., Barras & Kerzel, 2017; Duncan & Humphreys, 1989; Huurneman & Boonstra, 2015). While this did not directly relate to our primary outcomes, it could be a factor to consider in future studies.

The current study makes theoretical contributions to the exploration of the EQE. Lawrence and Pratt (2022) first changed the target-distractor similarity indirectly when investigating how the salience of the distractor influences the EQE. The current study, simultaneously and independently of Becker et al. (2025), provides direct evidence regarding the effect of target-distractor similarity on the EQE. Furthermore, the study offers additional insights by implementing different controls compared to those used by Becker et al. (2025). One difference is that the salient distractor in the current study had no delayed onset. Our findings suggest that this change did not influence the effect of target-distractor similarity on the EQE, supporting the findings of Lawrence et al., (2023a, 2023b) that delayed onset is not necessary for the EQE. Additionally, we used different set sizes in the task and examined the effect of target-distractor similarity across different experiments. Despite these changes, we found that the effect of target-distractor similarity was the same as the findings of Becker et al. (2025). Ultimately, the most important difference is that our explanation of the mechanism underlying the EQE places a different emphasis compared to Becker et al. (2025). Becker et al. (2025) employed a model to emphasize that the effect of target-distractor similarity leads to a rapid accumulation of information, influencing the decision-making process. Specifically, when the salient distractor does not share features with the target, it tends to suggest that the target is absent; whereas when the distractor shares features with the target, it tends to indicate that the target is present. However, we used specific psychological explanations to clarify what the information is and how it works differently. We stress that when there is no similarity between the salient distractor and the target, individuals find it easier to get the information that the distractor is not the target. This leads to quick attention suppression and a tendency to conclude that there is no target. It is this early-acquired information that results in early quitting of the search in target-absent trials and a higher error rate in target-present trials. When the salient distractor is partially similar to the target, individuals are less likely to immediately get the information about whether the distractor is the target or not, which leads to a longer time of attention suppression. As a result, the speed of quitting the search is slower, and no difference in error rates is observed for target-present trials.

To better understand real-world search, it is important to study the presence and characteristics of distractors, especially when the target is absent (Zhang, 2024). The present study highlights the practical implications of investigating the role of target-distractor similarity in target detection, particularly when the target is not always present. Moher (2020) has argued that the EQE may represent how salient the distractor influences search performance in real-world target-detection tasks. Given that it is mostly in an environment where objects are similar to each other we require the process of visual search like a laboratory search task, we tested the EQE when the target and distractor were similar to enhance the ecological validity and found that the EQE disappeared. Therefore, the impact of the EQE on real-world visual search is likely to be limited by target-distractor similarity. However, our manipulation involved presenting targets and distractors that shared the same orientation feature, but without introducing continuous differences in orientation. This raises the question of how far the similarity between the target and the distractor—specifically in terms of orientation—can influence the EQE. In other words, the precise boundary or extent of similarity that could reduce or eliminate the effects of the EQE is still unclear. Further investigation beyond the laboratory is needed in the future.

## Conclusion

The present findings provide new insight into how a salient distractor influences visual search. Specifically, when the similarity of the salient distractor and the target increased, a salient distractor induced delay quitting when searching in target-absent trials and had no effect on the error rate when searching in target-present trials. In other words, the early quitting effect disappeared. This study suggested that the impact of salient distractors in real-world target detection may be limited by the similarity between the target and the salient distractor.

### Significance statement

The present study investigates how the similarity between the target and salient distractor influences target detection in visual search tasks. This is particularly relevant for real-world scenarios, where distractors often resemble the target. For example, during baggage screening, harmless nontarget objects like metal hairpins can be confused with dangerous target items such as folding knives. The increased similarity of the target and salient distractor may thus enhance the ecological validity of the target-detection visual search tasks. In addition, this study can test the assumptions of underlying mechanisms of the early quitting effect. According to these assumptions, the increased similarity could either raise costs of attention suppression, leading to delayed response times in target-absent trials, or trigger the sense of effort in searching, resulting in the appearance of the early quitting effect. In the present study, we found that when a salient distractor shared no feature with a target, the presence of a salient distractor significantly shortened the search time when the target was absent (early quitting effect). However, when the salient distractor and target shared a feature, the salient distractor significantly delayed the search time. These findings suggest that attention suppression through the manipulation of similarity between the target and salient distractor, rather than the sense of effort, is the key modulator of the early quitting effect. Future research is needed to further explore these findings and their potential impact on real-world visual search tasks.

## Data Availability

The data in this study are available from the corresponding author upon reasonable request.

## Abbreviations

EQE: Early quitting effect
RT: Response time
